# Annexin A2 Contributes to Release of Extracellular Vimentin in Response to Inflammation

**DOI:** 10.1096/fj.202500793R

**Published:** 2025-05-10

**Authors:** Zhiyao Yuan, Z. Ostrowska‐Podhorodecka, T. Cox, M. Norouzi, Y. Wang, K. Robaszkiewicz, M. Siatkowska, K. Xia, A. Ali, M. Abovsky, I. Jurisica, P. Smith, C. A. McCulloch

**Affiliations:** ^1^ Nanjing Stomatological Hospital, Affiliated Hospital of Medical School, Research Institute of Stomatology Nanjing University Nanjing China; ^2^ Faculty of Dentistry University of Toronto Toronto Ontario Canada; ^3^ Department of Biochemistry and Cell Biology, Faculty of Natural Sciences Kazimierz Wielki University in Bydgoszcz Bydgoszcz Poland; ^4^ Laboratory of Molecular and Nanostructural Biophysics Bionanopark Lodz Poland; ^5^ Osteoarthritis Research Program, Division of Orthopedic Surgery, Schroeder Arthritis Institute and Data Science Discovery Centre for Chronic Diseases, Krembil Research Institute University Health Network Toronto Ontario Canada; ^6^ Department of Medical Biophysics University of Toronto Toronto Ontario Canada; ^7^ Department of Computer Science University of Toronto Toronto Ontario Canada; ^8^ Institute of Neuroimmunology Slovak Academy of Sciences Bratislava Slovakia; ^9^ Faculty of Medicine, School of Dentistry Pontificia Universidad Católica de Chile Santiago Chile

**Keywords:** Annexin A2, extracellular vimentin, periodontitis, secretion, unconventional protein secretion pathways

## Abstract

Vimentin, an abundant intracellular cytoskeletal protein, is secreted into the extracellular space, where it can amplify tissue destruction in inflammatory diseases. The mechanisms by which inflammation promotes the release of extracellular vimentin (ECV) are not defined. In human subjects, we found > twofold higher levels of ECV in gingival crevicular fluid from periodontitis sites with inflammation compared with healthy sites. In cultures of human gingival fibroblasts (hGFs) treated with 1% serum or IL‐1β (10 ng/mL) to model tissue injury or inflammation, respectively, we found that 1% serum increased ECV release > 11‐fold while IL‐1β further enhanced release 17‐fold. Mass spectrometry of vimentin immunoprecipitates identified Annexin A2 (AnxA2), a Ca^2+^‐dependent phospholipid‐binding protein, as a potential binding protein of ECV, which was confirmed by immunoprecipitation of cultured hGFs and immunostaining of inflamed human gingiva. IL‐1β treatment enhanced the abundance of AnxA2 and vimentin in membrane fractions prepared by sucrose gradients of hGF lysates. IL‐1β increased colocalization of ECV and AnxA2 at the outer aspect of the plasma membrane of intact hGFs. Knockdown of AnxA2 with siRNA or inhibition of the unconventional secretory pathway reduced ECV release from hGFs. These findings indicate that the production of ECV by hGFs in response to inflammation is mediated by an AnxA2‐dependent, unconventional secretory pathway that may play a role in amplification of the inflammatory response.

## Graphical abstract

The production of extracellular vimentin (ECV) by hGFs in response to periodontitis is mediated by an AnxA2‐dependent unconventional secretory pathway that may play a role in the amplification of the inflammatory response.

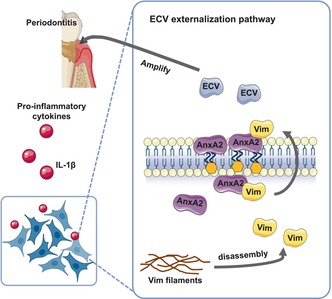

**Abbreviations**
AnxA2: Annexin A2
BAPTA/AM: 1,2‐bis (o‐aminophenoxy) ethane‐N,N, N',N'‐tetraacetic acid acetoxymethylester
BCA: bicinchoninic acid
BSA: bovine serum albumin
DAMP: damage associated molecular patterns
DMEM: Dulbecco's modified Eagle's Medium
ECV: extracellular vimentin
EDTA: ethylenediaminetetraacetic acid
EMT: epithelial–mesenchymal transformation
FBS: fetal bovine serum
GAPDH: glyceraldehyde 3‐phosphate dehydrogenase
GCF: gingival crevicular fluid
HCQ: hydroxychloroquine
hGF: human gingival fibroblast
HRP: horseradish peroxidase
IP: immunoprecipitation
LC3: microtubule‐associated proteins 1A/1B light chain 3B
MFI: mean fluorescence intensity
PBS: phosphate buffered saline
PFA: paraformaldehyde
PVDF: polyvinylidene fluoride
qRT‐PCR: quantitative reverse transcription polymerase chain reaction
RIPA: radioimmunoprecipitation assay
TLR4: toll like receptor 4
TMB: 3,3′,5,5′‐Tetramethylbenzidine
TNEV: 10 mM Tris/Cl, pH 7.5, 150 mM NaCl, 5 mM EDTA, 1 mM PMSF, 1 mM N_3_VO_4_
UPS: unconventional protein secretion

## 1 Introduction

Vimentin is an abundant, type III intermediate filament cytoskeletal protein in mesenchymal lineage cells such as fibroblasts and certain immune cells. Vimentin assembles into filaments in a hierarchical manner to maintain the structural integrity of connective tissue cells [1, 2] and is implicated in several processes relevant to inflammatory diseases such as epithelial–mesenchymal transition [3], cell migration [4, 5, 6], and tissue remodeling [7]. In pathological conditions such as inflammation, tissue injury, or autoimmune disease, vimentin undergoes posttranslational modifications (PTMs) including phosphorylation and citrullination, which promote the disassembly of vimentin filaments into soluble oligomers. When released by fibroblasts, endothelial cells, immune cells, or cancer cells [1, 8, 9, 10], soluble forms of vimentin are secreted as extracellular vimentin (ECV). ECV can enter the circulation [1, 11, 12] and is detectable in bodily fluids such as cerebrospinal fluid [13] and urine [14]. Elevated ECV levels are present in the blood of patients with systemic inflammation or coronary artery disease [15, 16], and in the fluid draining from localized inflammatory lesions such as periodontitis [17]. ECV can also act as damage‐associated molecular patterns that amplify immune responses, as seen in periodontitis [18].

Periodontitis is a chronic inflammatory disease of tooth‐supporting tissues caused by interactions between tooth‐borne microbial biofilms and the host immune system [19]. Destruction of periodontal tissues in periodontitis reduces oral function and is the major cause of tooth loss in adults. Beyond its impact on oral health and quality of life, periodontitis is associated with increased risk of certain systemic diseases such as diabetes, myocardial infarction, and rheumatoid arthritis, which are important socioeconomic burdens [20]. During the development of periodontitis, the host immune system responds to the invasion of pathogens in periodontal tissues by recruiting immune cells. One of these cell populations is the macrophage, which releases pro‐inflammatory mediators (e.g., IL‐1β) [21, 22, 23] into affected sites that promote destruction of periodontal connective tissues [24, 25].

Mass spectrometry analysis of human gingival crevicular fluid (GCF) from periodontitis sites shows increased abundance of vimentin peptides compared with healthy sites [17] but these findings have not been confirmed by western blotting. While ECV release from endothelial cells is in part mediated by unconventional protein secretion (UPS) [26], the mechanism by which vimentin is transported across the plasma membrane and secreted by periodontal cells is not known.

Annexin A2 (AnxA2) is a prominent plasma membrane protein that binds vimentin [27] and is abundant in GCF draining from inflamed human gingiva [28]. Notably, AnxA2 does not contain a signal peptide. The translocation of AnxA2 to the external leaflets of the plasma membrane is well defined but may involve an UPS pathway and is associated with exosomal secretion, similar to that of vimentin [26]. The N‐terminal domain of AnxA2 contains four annexin repeats that mediate Ca^2+^‐dependent binding to phospholipids [29, 30, 31]. In the presence of Ca^2+^, AnxA2 undergoes a conformational change that exposes its N‐terminal domain. This domain contains a binding site for S100A10, a Ca^2+^‐binding protein that forms a hetero‐tetrameric complex with AnxA2. This complex regulates the secretion of AnxA2 and its bound proteins [29, 32, 33]. Notably, macrophage inflammatory protein‐1α/β can induce the translocation of AnxA2 from the cytoplasm to the external surface of the plasma membrane [34]. In this report, we describe the effect of inflammation and the role of the AnxA2‐dependent UPS pathway in ECV generation.

## 2 Materials and Methods

### 2.1 Reagents and Antibodies

The following antibodies were purchased from Proteintech (Rosemont, IL): anti‐vimentin monoclonal (Mouse/IgG1, # 60330‐1‐Ig), anti‐vimentin polyclonal (Rabbit/IgG, #10366‐1‐AP), anti‐AnxA2 monoclonal (Mouse/IgG2a, # 66035‐1‐Ig), and anti‐GAPDH monoclonal (Mouse/IgG2b, # 60004‐1‐Ig). Recombinant human AnxA2 (#230‐30 023‐100), derived from transfected human HEK293 cells, was obtained from RayBiotech (Peachtree Corners, GA). ON‐TARGETplus Non‐targeting Control Pool (5 nmol) and ON‐TARGETplus siRNA Reagents‐ AnxA2 5 nmol (SMARTpool format) were from Horizon (Lafayette, CO).

### 2.2 GCF Collection

GCF samples were obtained with periodontal paper strips (0–1.2 μL of fluid; Oralflow, NY). A calibrated periodontal specialist evaluated the periodontal health status in patients presenting for periodontal treatment at the University Clinical Center at the School of Dentistry, Pontificia Universidad Católica de Chile. None of the subjects had received periodontal treatment or had consumed systemic antibiotics during the previous 6 months prior to sample collection. No diabetics, smokers, or subjects with chronic systemic diseases such as rheumatoid arthritis were included in the study. Perio paper strips were inserted subgingivally for 30 s at selected sites. Blood‐contaminated perio paper strips were discarded. The human experimentation protocol for sampling was approved by the institutional review board of Pontificia Universidad Católica de Chile (ID: 210615009). All subjects provided informed consent before GCF sampling.

### 2.3 ECV Detection in GCF Samples

After sampling, paper strips were eluted in Eppendorf tubes containing 20 μL of PBS plus a “cocktail” of protease inhibitors (Sigma‐Aldrich). Samples were sedimented to remove cells and stored in liquid nitrogen for up to 72 h. After defrosting, samples were mixed with 1x Laemmli loading buffer and boiled at 98°C for 5 min. Western blotting and densitometry were used to quantify the amount of vimentin with an anti‐vimentin antibody (ProteinTech, 10366‐1‐AP, 1:2000). Vimentin abundance was normalized to the abundance of total proteins using Ponceau Red staining (ThermoFisher, A40000279). Cell lysates prepared from human gingival fibroblasts were used as a positive control for vimentin in western blots.

### 2.4 Analysis of Immunostained Gingiva

Human gingival biopsies were obtained from adult subjects (41–62 years of age) undergoing periodontal surgical procedures, which included tissues from healthy (*n* = 6; 3 females, 3 males) and inflamed (*n* = 7; 4 females, 3 males) sites. Biopsies were obtained under University of Toronto Research Ethics Board protocol #32822. Tissues were fixed in 4% paraformaldehyde for 1 day, washed, and embedded in paraffin for preparation of sagittal sections (5 μm). Sections were prepared and stained with H&E for histological evaluation. Gingivitis was categorized on the basis of the inflammatory infiltrate as mild, moderate, or severe based on examination of the H&E‐stained slides [35]. For immunofluorescence examination, sections were deparaffinized and then immunostained for vimentin and AnxA2. Sections were counterstained with DAPI as described [36] and imaged by confocal microscopy (Zeiss LSM 800) using ×10 and ×40 objectives with image processing by Zen Blue (Zeiss).

### 2.5 Cell Culture and Transfection

Human gingival fibroblasts (hTERT‐HGF‐CRL‐4061‐ATCC) were cultured in complete DMEM supplemented with 1% and 10% fetal bovine serum (FBS), penicillin (100 U/mL), and streptomycin (100 μg/mL) at 37°C. To knockdown AnxA2, cells were transfected with the corresponding siRNA using Lipofectamine 3000. Controls were treated with the nontarget control Dharmacon ON‐TARGET plus transfection reagents.

### 2.6 Western Blotting

Protein was extracted from cells with RIPA buffer supplemented with protease and phosphatase inhibitors. Protein concentration was determined using the BCA assay according to the manufacturer's protocol. Equal amounts of protein were separated on SDS‐PAGE gels and transferred onto PVDF membranes. The membranes were blocked with BSA in Tris‐buffered saline with 0.1% Tween‐20 (TBST) at room temperature for 1 h and incubated with primary antibodies overnight at 4°C. After washing with TBST, the membranes were incubated with fluorescent‐conjugated secondary antibodies for 1 h at room temperature. Protein bands were visualized using an enhanced chemiluminescence detection kit.

### 2.7 Quantitative Reverse Transcription Polymerase Chain Reaction (qRT–PCR)

RAW264.7 cells were seeded and incubated in complete DMEM until the day of treatment. Cells were then stimulated for 16 h with either PBS (control), 10 ng/mL, or 100 ng/mL of purified vimentin in DMEM containing 1% FBS. Total RNA was extracted and 1 μg of total RNA was used for reverse transcription in a 20 μL reaction volume. The resulting cDNA was diluted 1:10, and 5 μL was used per qPCR reaction, which was performed in triplicate. IL‐1β mRNA expression was analyzed using quantitative real‐time PCR with the following primers: IL‐1β: Forward 5′‐CCTTCCAGGATGAGGACATGA‐3′, Reverse 5′‐TGAGTCACAGAGGATGGGCTC‐3′; GAPDH: Forward 5′‐CCTTCCGTGTTCCTACCCC‐3′, Reverse 5′‐GCCCAAGATGCCCTTCAGT‐3′. mRNA expression levels were calculated using the 2^−ΔΔCt^ method, with GAPDH as the reference gene for normalization.

### 2.8 Membrane Protein Analysis

Cells (3 × 10^7^–1 × 10^8^) were collected by centrifugation at 250 *g* for 7 min and washed twice with ice‐cold PBS. Cell pellets were resuspended in 1.4 mL ice‐cold 1% Triton X‐100 in 1 mM PMSF Tris Sodium EDTA vanadate buffer and incubated on ice for 1 h. Lysates were homogenized by passing through a 10 μL pipette tip 10–15 times and centrifuged at 700 *g* for 8 min at 4°C. Supernatants were collected and mixed with an equal volume of 85% sucrose in TNEV buffer, then overlaid with 35% and 5% sucrose layers to form a discontinuous sucrose gradient. Ultracentrifugation was performed at 38 000 rpm (257 000× *g*) for 18 h at 4°C in an SW40 rotor. Fractions (1 mL each, 12 in total) were collected from the top of the gradient. Plasma membrane fractions were identified at the interface between 5% and 35% sucrose, while flow‐through fractions represented non‐lipid raft components. Plasma membrane and flow‐through fractions were analyzed by western blot to detect proteins that were anticipated in these fractions.

### 2.9 Protein Structure Prediction and Docking

Molecular docking analysis was performed using AlphaFold monomer V2.0 predictions for Annexin A2 (P07355) and Vimentin (P08670). ChimeraX software ver. 1.7.1 (2024‐01‐23) was employed for docking preparation and visualization. The highest probability docking poses were computed on publicly available web servers (accessed September 22–24, 2024): ClusPro (https://cluspro.bu.edu), LZerD (https://lzerd.kiharalab.org), LightDock (https://server.lightdock.org), and a freely available MEGADOCK v.4.1.1. software (https://www.bi.cs.titech.ac.jp/megadock/ppi.html).

### 2.10 Measurement of AnxA2‐Vimentin Interaction

An ELISA plate (Corning #9017) was coated with vimentin (Vim‐coated) or PBS (Blank) for 1 h at room temperature (RT). The plate was washed and blocked with blocking buffer (5% NFDM) for 1 h at RT. After a wash step, AnxA2 was added at 1.5 μM and 0 μM to Vim‐coated wells and at 1.5 μM to Blank wells and incubated for 1 h at RT. Anti‐His tag primary antibody (Invitrogen #MA1‐21315) was added at a 1:1000 dilution, and the plate was incubated for 1 h at RT. After a wash step, HRP‐conjugated secondary antibody (CST #91196) at a 1:1000 dilution was added for 30 min at RT followed by a wash step. 3,3′,5,5′‐Tetramethylbenzidine (TMB) substrate (Thermo Scientific #34028) was added to wells and incubated for 5 min at RT, and the reaction was quenched by the addition of sulfuric acid. AnxA2‐Vimentin interaction was assessed by measuring plate absorbance at 450 nm.

### 2.11 Immunoprecipitation

After overnight culture on 100 mm culture dishes, cells were lysed in IP lysis buffer (25 mM Tris–HCl pH 7.4, 150 mM NaCl, 1 mM EDTA, 1% NP‐40, and 5% glycerol) supplemented with 1 mM phenylmethylsulphonyl fluoride, 1 mM NaVO3, 10 μg/mL leupeptin, and 10 μg/mL aprotinin. Equal amounts of protein from the cleared extracts were used for immunoprecipitation using Dyna‐beads Protein A (Life Technologies) according to the manufacturer's protocol (Invitrogen, Carlsbad, CA) with specific primary antibodies (vimentin or AnxA2) or with pre‐immune serum. Beads were separated magnetically and washed twice with IP buffer before resuspension in 2× Laemmli sample buffer and boiled for 10 min. Proteins were eluted from beads, and cleared lysates were immunoblotted or analyzed by mass spectrometry.

### 2.12 Mass Spectrometry

Vimentin immunoprecipitates using beads coated with antibodies to vimentin or immunoprecipitates prepared from beads coated with pre‐immune serum were eluted from beads and digested with 800 ng per sample of trypsin (Pierce) at 37°C overnight. Peptide extraction was done with 5% formic acid and 100% acetonitrile, and lyophilization with a Speedvac. Pellets were resuspended in 2% acetonitrile and 0.1% formic acid. Mass spectrometry (SPARC Biocentre, Hospital for Sick Children, Toronto, ON) was conducted with a Thermo Scientific Orbitrap Fusion Lumos mass spectrometer in tandem with an Evosep One liquid chromatography system using a C18 AQ column. Chromatography was conducted with 1.9 μm beads in a column of 150 μm ID and 15 cm length. Buffer A consisted of 0.1% formic acid and buffer B contained 100% acetonitrile and 0.1% formic acid. All protein identification and quantification were conducted with Proteome Discoverer 2.5.0.400 and Scaffold 5.0.1. For assessing proteins that are associated with vimentin, the peptides that were identified with beads coated with pre‐immune serum were subtracted from the peptides identified with beads coated with an antibody to vimentin.

### 2.13 Immunostaining

Cells were cultured on sterilized glass coverslips and washed twice with PBS. For immunostaining of permeabilized cells, cells were fixed with 4% paraformaldehyde for 15 min at room temperature, permeabilized with 0.1% Triton X‐100 in PBS for 10 min, and blocked with 5% BSA in PBS for 30 min to reduce nonspecific binding. Coverslips were incubated with primary antibodies for AnxA2 or vimentin (diluted in 1% BSA/PBS) at 4°C overnight. After washing three times with PBS, cells were incubated with fluorescence‐conjugated secondary antibodies (Alexa Fluor 488 or Alexa Fluor 594) at room temperature for 1 h in the dark. Nuclei were counterstained with DAPI (1 μg/mL) for 5 min. For non‐permeabilized cells, samples were immunostained with antibodies, then fixed by PFA and stained with DAPI. Coverslips were mounted on glass slides with anti‐fade mounting medium and imaged with a confocal microscope (Zeiss LSM 800).

### 2.14 Autophagic Flux

We estimated autophagic flux in cells that were serum‐deprived or treated with 1% serum. We measured LC3 processing by western blot as described [37]. The rationale for this approach is that LC3 is conjugated to the inner surface of the autophagosome upon delivery to lysosomes for protein degradation. Detection of LC3 conjugation by western blot provides quantitative measures of bulk autophagic flux, which could be stimulated by serum depletion. HGFs were serum‐deprived for 6 h or incubated with 1% FBS. We measured LC3B‐I/II levels with and without an inhibitor of lysosomal degradation (hydroxychloroquine, HCQ; 50 mM). The amount of LC3B‐II delivered to the lysosome, measured as the increase in LC3B‐II when lysosomal degradation is inhibited for each condition, reflects the amount of ongoing autophagic flux. HGF lysates were immunoblotted for LC3B I and II, which are detected as 15 and 12 kDa bands, respectively.

### 2.15 Statistical Analysis

Statistical significance (*p* < 0.05) of differences between two groups was assessed using the Student's *t*‐test or analysis of variance for more than two groups and employed three biological replicates in each group. For analysis of the spatial relationship of proteins in cultured cells, Manders correlation analysis was used. Analyses were performed with GraphPad Prism 9 software. All experiments, except as indicated, were performed independently in triplicate on separate days. Histograms display means and SEM.

## 3 Results

### 3.1 ECV in GCF Increases in Periodontitis and Further Increases the Inflammatory Response

**FIGURE 1:**
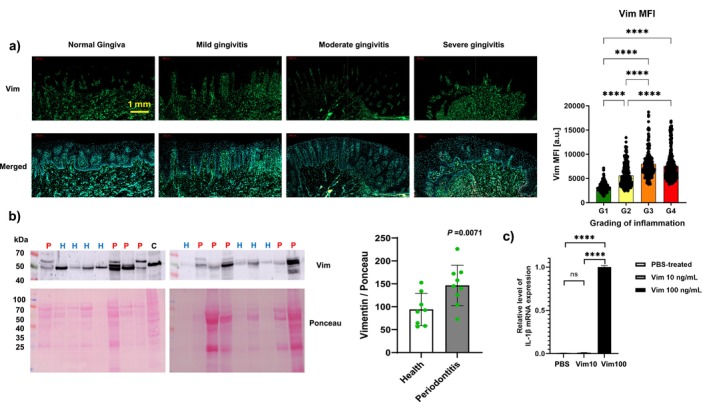
(a) Immunostaining of vimentin in normal gingiva (G1), mild gingivitis (G2), moderate gingivitis (G3) and severe gingivitis (G4). Differences between group means of fluorescence intensity (MFI) were assessed by analysis of variance and Tukey's test. Statistical differences are shown as: *p* < 0.0001. (b) ECV detection by western blot in gingival crevicular fluid obtained from periodontitis (P) and healthy (H) sites. An hGF lysate (C) was used as a positive control for vimentin. Quantification of western blot and Ponceau staining was expressed as a ratio of their abundance (Vimentin/Ponceau). (c) IL‐1β mRNA expression in RAW264.7 macrophages treated with PBS (control), 10 ng/mL vimentin, or 100 ng/mL vimentin. The means of these data were compared with Student's *t*‐test. Statistical differences are shown as: ****p < 0.0001; ns, no significance.

As the proteins in GCF, a transudate, originate largely from resident cells in the gingival lamina propria of sampled sites [38], we immunostained vimentin in biopsies of normal gingiva (G1) and biopsies with histological features of mild (G2), moderate (G3), and severe (G4) gingival inflammation. The mean fluorescence intensity of immunostained vimentin increased progressively with the different stages of inflammation (i.e., G1 < G2 < G3 < G4; Figure 1a), which reflected the higher numbers of vimentin‐expressing cells, including fibroblasts [39], in inflamed gingiva. We immunoblotted GCF samples from periodontitis sites (P; *n* = 9) and healthy sites (H; *n* = 8) for ECV and compared the molecular mass of vimentin with cell lysates prepared from hGFs as a positive control (C). The abundance of ECV (54 kDa) was normalized to the total protein in each sample lane, which was estimated by Ponceau Red staining of the corresponding lane of the nitrocellulose membranes used for immunoblots. By densitometry, ECV levels estimated by immunoblot were > twofold higher in GCF samples from periodontitis sites than healthy sites (*p* < 0.01; Figure 1b), which is in agreement with earlier data using mass spectrometry analysis of vimentin peptides found in GCF [17].

To assess the role of ECV in inflammation, we purified vimentin protein and stimulated RAW264.7 with different concentrations of vimentin. qPCR analysis revealed that IL‐1β expression was barely detectable in PBS‐treated RAW264.7 cells. After stimulation with 10 ng/mL vimentin, there was no increase in IL‐1β expression, while treatment with 100 ng/mL vimentin strongly upregulated IL‐1β expression, indicating that ECV enhanced IL‐1β expression in macrophages (Figure 1c). These results are consistent with previous studies [16] demonstrating that ECV can induce an inflammatory phenotype in immune cells. Accordingly, by amplifying IL‐1β expression, ECV may serve as a DAMP that promotes inflammation in periodontitis. We also considered whether ECV may activate hGFs by increasing the expression of toll‐like receptor 4 (TLR4), as has been demonstrated recently for neutrophils [40]. Under the experimental conditions that we used (Figure S1), treatment of hGFs with 10 or 100 ng/mL overnight did not increase the abundance of TLR4 mRNA compared with controls.

### 3.2 Vimentin Secretion in Response to Injury and Inflammatory Stimuli

**FIGURE 2:**
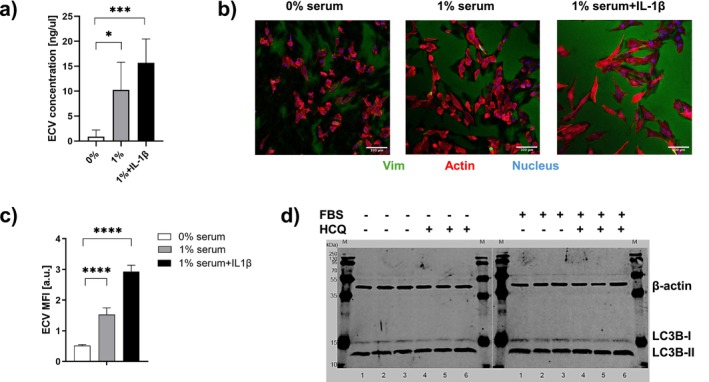
(a) The estimated concentration of ECV secreted into the culture media based on Western blot, densitometry of vimentin bands and comparison of experimental bands with known vimentin standards. (b, c) Immunofluorescence staining and quantification of ECV deposition on to culture substrate. (d) Potential contribution of autophagy in serum depletion. hGFs were serum‐deprived for 4 h (left blot) or incubated with 1% FBS (right blot). LC3B I and II (15 and 12 kDa). Cells were treated with or without hydroxychloroquine (HCQ; 50 μM; indicated as +). Cell lysates were examined by immunoblotting. Equality of protein loading was done with β‐actin. Data in panels a and c are means±standard errors. Differences between group means were assessed by analysis of variance and Tukey's test. Statistical differences are shown as: *p < 0.05; ***p < 0.001; ****p < 0.0001.

We examined whether the secretion of vimentin and its presence in GCF could be modeled by culture of hGFs and collection of ECV released by cells into the culture medium. Cells were treated with IL‐1β (10 ng/mL), a pro‐inflammatory cytokine implicated in the progression of periodontitis [41] or with 1% FBS, used here as a surrogate for tissue injury [42]. Compared with cells cultured in serum‐free medium, cells incubated for 16 h in medium supplemented with 1% FBS showed a robust increase of ECV secretion (~11.4‐fold; *p* = 0.0102). The increase of ECV secretion relative to serum‐free conditions was also replicated and increased by IL‐1β (~17.4‐fold; *p* < 0.0001; Figure 2a). We also assessed ECV deposition around cells by immunostaining non‐permeabilized cells and their surrounding substratum (Figure 2b) as previously described [26]. Quantification of mean fluorescence intensity (MFI) attributable to vimentin attached to the substrate indicated that, compared with controls, ECV deposition was associated with injury and inflammatory stimulation (Figure 2c).

As nutrient deprivation can in certain cell types promote autophagy‐induced release of intracellular proteins [43], and possibly vimentin, we considered the possibility that ECV secretion in these conditions may reflect autophagic activation. For assessing autophagy, we measured the processing of LC3 [37], a protein that is conjugated to the inner surface of autophagosomes delivered to lysosomes for degradation. Hydroxychloroquine, the hydroxylated analog of chloroquine, is a weak base that inhibits autophagy due to lysosomal acidification and subsequently blocks the fusion of autophagosomes with lysosomes. hGFs were serum‐deprived or incubated with 1% FBS (Figure 2d, right blot). LC3B‐I/II levels were measured in cells treated with or without hydroxychloroquine (HCQ; 50 μM) [44]. The amount of LC3B‐II delivered to lysosomes, which is reflected by the increase in LC3B‐II levels when lysosomal degradation is inhibited, indicates ongoing autophagic flux. We did not observe a difference in autophagic flux between 1% and 0% FBS conditions, indicating that ECV secretion observed under these conditions is independent of alterations in lysosome function related to autophagy.

### 3.3 Vimentin Interacts With the Phospholipid‐Binding Protein AnxA2

**FIGURE 3:**
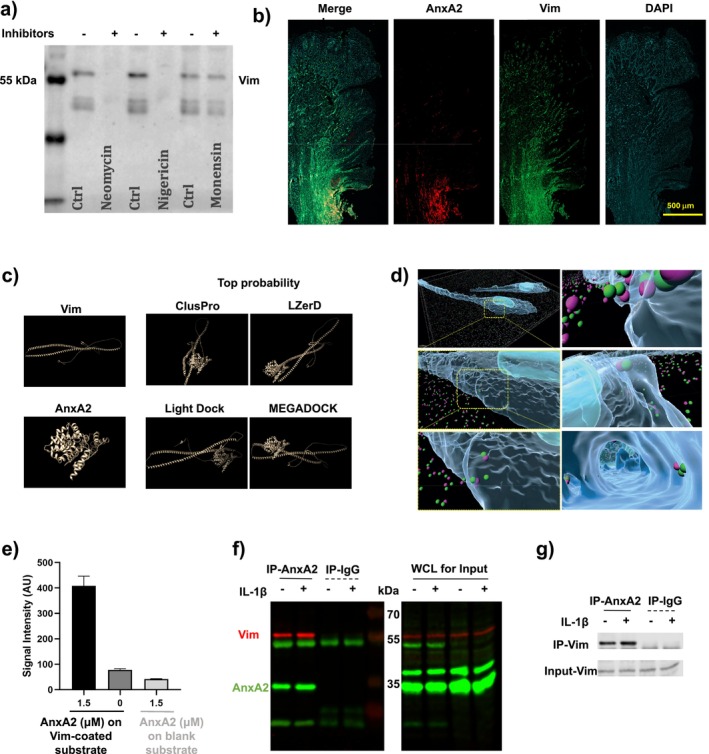
(a) Secretion of ECV was unaffected in hGFs treated with the Golgi‐dependent transport blocker (monensin) compared with controls while an inhibitor of the UPS pathway (nigericin) and an inhibitor of phosphoinositide‐related UPS (neomycin) caused a reduction in ECV levels compared with their controls. (b) Immunofluorescence staining of sagittal sections through inflamed human gingiva show colocalization of vimentin (green) and AnxA2 (red). (c) AlphaFold v.2.0 structures of AnxA2 and Vim were used for analysis by ChimeraX software for docking preparation and graphical presentation. The docking poses were computed using MEGADOCK software and ClusPro, LZerD, or LightDock web servers. (d) Immunostaining followed by image analysis using Imaris shows spatial association of ECV (green dots) and AnxA2 (violet dots) in cultured hGFs. (e) The AnxA2‐Vimentin protein interaction was assessed by ELISA. (f, g) Interaction of AnxA2 with vimentin was assessed by immunoprecipitation of HGF lysates. Cells were treated with or without IL‐1β (10 ng/mL) for 30 min prior to cell lysis. Immunoprecipitations were conducted with AnxA2 antibody (IP‐AnxA2) or with pre‐immune serum (IP‐IgG). Panel (f) shows AnxA2 immunoprecipitates that were western blotted for vimentin (red) and for AnxA2 (green). The whole cell lysate (WCL) panel on the right shows immunoblots of the four different cell preparations that were immunoprecipitated in the left panel. Panel (g) shows the gray‐scale western blot analysis for the vimentin in the four cell preparations.

For obtaining insight into vimentin secretion, we screened for the effects of regulators of cell secretion on ECV. Based on earlier methods and optimized concentrations of inhibitors used for examining endothelial cell secretion of ECV [26], we examined the impact of the Golgi‐dependent transport blocker (monensin, 25 nM), the H^+^ and K^+^ ionophore nigericin (25 μM), which inhibits UPS [26], and an inhibitor of the phosphoinositide‐related UPS pathway (neomycin, 10 mM). In contrast with HCQ (see above), treatments that affected the efficacy of the UPS pathway (neomycin and nigericin), but not the Golgi‐dependent pathway blocker monensin, reduced the concentration of ECV in the culture medium compared with controls (Figure 3a). These data indicate that UPS pathways may contribute to vimentin secretion in hGFs. Consequently, as vimentin lacks a signal peptide required for the classical ER‐Golgi secretion pathway, we explored molecular partners and secretory machinery involved in vimentin transport across the plasma membrane.

By mass spectrometry analysis of vimentin immunoprecipitates prepared from HGF lysates in four replicated experiments, we identified potential molecules that associated with vimentin (Table 1). Specifically, we searched for relatively high‐abundance plasma membrane‐associated proteins [45, 46] that are involved in the UPS pathway and that are also linked to gingival inflammation. Based on analysis of peptide counts and a recent report demonstrating that AnxA2 abundance is increased in GCF from inflamed periodontal sites [28], we considered that AnxA2 was a high‐likelihood candidate that fulfilled these conditions. In addition, S100A10 was identified as a vimentin‐binding protein, although at lower peptide counts than AnxA2. As S100A10 forms a heterotetrameric complex with AnxA2 [47], its association suggests a potential role in vimentin secretion although we have not pursued this potential role further. Colocalization of vimentin and AnxA2 in the lamina propria was observed by immunostaining of inflamed gingival tissue (Figure 3b), suggesting a potential interaction between these two proteins. We explored this association with AlphaFold modeling and docking analyses with four different algorithms. These analyses indicated a high probability of a direct interactions between AnxA2 and vimentin (Figure 3c). To validate these predictions at the cellular level, immunostaining of non‐permeabilized hGFs followed by confocal imaging and Imaris analysis of the plasma membrane's external surface demonstrated a moderate level of spatial association between ECV and AnxA2 (Figure 3d). To confirm direct binding between AnxA2 and vimentin, an ELISA assay was performed. The signal intensity of purified AnxA2 (1.5 μM) when incubated with purified vimentin‐coated substrate was higher than that of both the 0 μM AnxA2 on vimentin‐coated substrate group and the 1.5 μM AnxA2 on blank substrate, confirming an authentic AnxA2‐vimentin interaction (Figures 3e and S2). Finally, immunoprecipitation experiments of intact cells were conducted that supported this interaction in hGFs and that showed an increased association between AnxA2 and vimentin after IL‐1β treatment compared to vehicle controls (Figure 3f,g).

**TABLE 1:** Mass spectrometry analysis of high probability vimentin associating proteins.

Protein	Peptide counts			
	Experiment 1	Experiment 2	Experiment 3	Experiment 4
	Mean	Mean	Mean	Mean
Vimentin	93.50	40.00	89.70	576.60
Plectin	340.50	186.00	317.50	1836.10
Myosin‐9	208.00	113.00	198.90	537.10
Galectin‐3	3.25	3.00	3.60	3.60
Ezrin	20.25	13.50	37.70	76.30
Catenin beta‐1	13.00	9.00	10.40	13.50
Cofilin‐1	9.25	16.00	10.89	12.30
Moesin	58.50	30.50	2.00	163.20
**Annexin A2**	**53.00**	**24.00**	**49.20**	**277.60**
Annexin A1	19.50	21.50	26.40	39.70
ATP‐binding cassette subfamily E member 1	11.00	11.00	6.10	14.00
ATP‐binding cassette subfamily F member 2	12.25	13.00	11.20	13.40
ATP‐binding cassette subfamily D member 3	5.00	8.00	4.90	5.60
ATP‐binding cassette subfamily F member 3	3.75	4.00	3.90	4.20
Protein S100‐A10	9.75	3.00	16.00	35.20
Protein S100‐A11	7.75	3.00	9.20	32.40
Protein S100‐A4	9.75	2.00	14.20	22.50
Protein S100‐A13	6.50	2.00	6.80	10.40
Protein S100‐A16	6.50	6.80	6.20	9.90
Protein S100‐A6	5.00	4.00	5.00	7.60

*Note:* Data in table are mean peptide counts from four independent experiments. Only peptide counts with high probability of correct identification of proteins are included (*p* < 0.001). The values shown in the table were obtained by subtracting the peptide counts from pre‐immune serum‐coated beads from the peptide count of beads coated with vimentin antibodies. Mass spectrometry hit for annexin A2 are highlighted in bold.

### 3.4 Colocalization of ECV and AnxA2 in the Plasma Membrane

**FIGURE 4:**
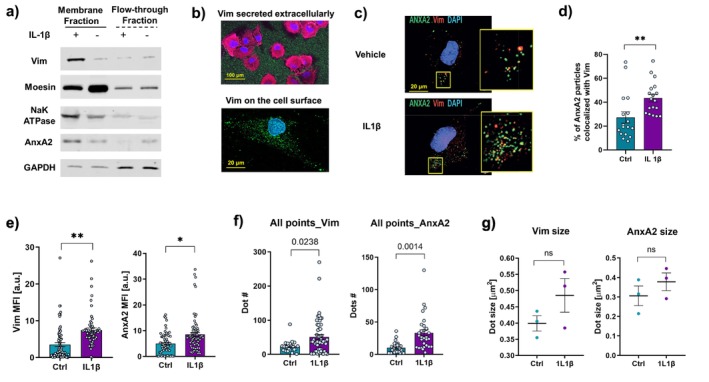
(a) HGFs were treated with IL‐1 (lanes 1, 3) or vehicle (lanes 2, 4). Plasma membrane preparations (lanes 1, 2) were selectively enriched with the plasma membrane associated proteins moesin and Na, K‐ATPase. Flow‐through fractions (lanes 3, 4) were enriched with the Triton‐X soluble protein GAPDH. Vimentin and AnxA2 were more abundant in the IL‐1‐treated sample. (b) No permeabilization of the hGFs was done until after immunostaining to exclude inadvertent staining pf intracellular vimentin, which enabled specific labeling of ECV, especially ECV on the cell surface. (c) Immunostaining for ECV and AnxA2 on the most exterior portion of the plasma membrane of non‐permeabilized fibroblasts. (d) Extent of spatial colocalization of AnxA2 and ECV shows higher colocalization (Manders) after IL‐1β treatment. (e–g) The mean fluorescence intensity (MFI in arbitrary units) for vimentin and AnxA, the number and mean size of discrete ECV and AnxA2 dots on the plasma membrane were quantified by Imaris in 30 different cell preparations. All points > 1 μm in diameter that were immunostained for AnxA2 or vimentin were quantified per cell in a 40× objective optical field. Data are means and differences between groups compared with Student's t‐test are shown with *p < 0.05, **p < 0.01 and ns, no significance.

AnxA2 is a phospholipid‐binding protein that is enriched in plasma membranes and facilitates membrane‐related processes such as membrane repair and the attachment of secretory granules to the plasma membrane during exocytosis [48]. We considered that AnxA2 spatially associates with vimentin, facilitates its localization to the plasma membrane, and enables extracellular release by translocation to the cell exterior [34]. To examine this possibility, hGFs were treated with IL‐1β or vehicle (30 min; 10 ng/mL) and lysed. Plasma membrane fractions were prepared with sucrose gradients. Plasma membrane fractions (lanes 1,2; Figure 4a) were selectively enriched with the plasma membrane proteins moesin, AnxA2, and Na/K‐ATPase. ECV and AnxA2 were more abundant in IL‐1β‐treated samples. Flow‐through fractions (lanes 3,4) were enriched with the Triton‐X soluble protein GAPDH but not with AnxA2 or ECV.

For specific labeling of ECV that would avoid detection of intracellular vimentin, hGFs were not permeabilized prior to immunostaining. After immunostaining, cells were stained with DAPI or for actin filaments. With this method, after washing, ECVs on the cell surface could be readily observed under high magnification (Figure 4b). For assessing the localization of ECV and AnxA2 in the plasma membrane, live, non‐permeabilized cells were immunostained with vimentin and AnxA2 antibodies. This procedure was followed by fixation and nuclear staining with DAPI. As described above, this method excluded staining of intracellular vimentin filaments and intracellular AnxA2; only molecules on the surface of the plasma membrane were labeled. Confocal microscopy showed colocalization of ECV and AnxA2 on the plasma membrane as yellow dots, which was further enhanced by IL‐1β treatment (Figure 4c–g). These findings suggest that in response to inflammation, the secretion of vimentin in a direction toward the plasma membrane surface is increased and that AnxA2 may play a role in this process. Further, we also examined whether the vimentin and AnxA2 dots observed on the cell surface were also visible on the substrate, as suggested in the low power images in Figure 2C. High power magnification confocal images showed similar sized dots of vimentin and AnxA2 (Figure S3) as quantified in Figure 4.

### 3.5 Release of Vimentin From hGFs in an AnxA2‐Dependent Manner

**FIGURE 5:**
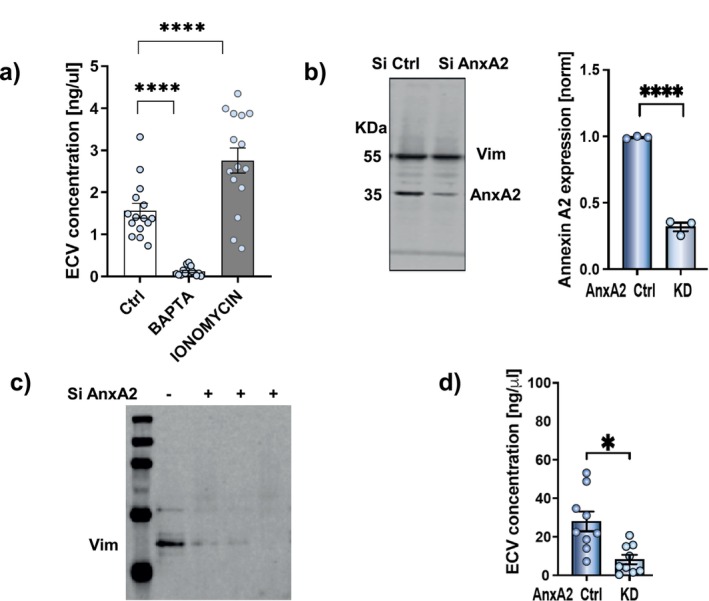
(a) Vimentin secretion is regulated by [Ca^2+^]_*i*_ concentration changed by calcium chelator BAPTA‐AM (10 μM) and ionomycin (Ca^2+^ ionophore). (b) siRNA knockdown of AnxA2 but with no effect on intracellular vimentin abundance as measured by western blot. (c, d) Measurement of ECV released into fibroblast culture medium after control siRNA (−) or siRNA knockdown of AnxA2 (+; 3 u = different samples). Statistical differences are shown as: *p < 0.05; ****p < 0.0001.

Since the interaction of AnxA2 with other associating proteins is Ca^2+^‐dependent [49], we examined the impact of varying [Ca^2+^]_*i*_ on ECV secretion. These analyses indicated that vimentin passage through the plasma membrane and the resultant production of ECV is regulated by [Ca^2+^]_*i*_ (Figure 5a). Treatment with the intracellular calcium chelator BAPTA/AM (10 μM) inhibited vimentin secretion (*p* < 0.0001) compared with the control group. Conversely, ionomycin (a Ca^2+^ ionophore; used here at 0.25 μM) promoted ECV secretion (*p* < 0.0001).

For more specific assessment of the role of AnxA2 in vimentin secretion, we used siRNA to knock down AnxA2 expression in hGFs. After siRNA treatment of AnxA2, compared with a siRNA control, the levels of AnxA2 were reduced by ~70% while the total vimentin expression remained unchanged (Figure 5b). We examined vimentin secretion after AnxA2 knockdown and found that ECV secretion was reduced by > 70% (Figure 5c,d). Collectively, these results indicate a role for AnxA2 in facilitating the transport of ECV.

## 4 Discussion

Our main finding is that ECV release is regulated by an AnxA2‐dependent pathway, which is enhanced by inflammatory stimuli such as IL‐1β. This finding advances our understanding of the mechanisms underlying ECV secretion and highlights a potential therapeutic target for mitigating inflammation‐driven tissue damage in periodontal disease. Vimentin is an abundant intracellular cytoskeletal protein that is secreted extracellularly as ECV under pathological conditions such as inflammation and tissue injury [50]. Elevated ECV levels have been implicated in amplifying the inflammatory response and promoting tissue destruction in various diseases, including periodontitis [18]. A previous mass spectrometry analysis [17] showed elevated ECV levels in GCF from periodontitis sites compared with healthy controls. Our quantitative western blot data are in agreement with these earlier findings of ECV in GCF and suggest that poorly controlled ECV release is associated with the inflammatory response and tissue damage in periodontitis, as previously described for other inflammatory and autoimmune diseases [18]. In periodontitis, poorly controlled inflammatory responses often lead to destruction of periodontal tissues, which may be exacerbated by ECV citrullination and is manifest as marked alveolar bone resorption and collagen degradation [51].

Unlike the classical protein secretion pathway, which involves trafficking of nascent proteins through the endoplasmic reticulum and the Golgi apparatus, proteins secreted via UPS pathways bypass these organelles. UPS pathways are used for secretion of proteins that lack a transmembrane domain or a signal peptide and are often triggered by stressors such as infection or inflammation [52, 53]. As vimentin lacks a signal peptide, the secretion of ECV via UPS pathways is a reasonable notion. In endothelial cells, recent data [26] indicate that ECV is secreted through the Type III UPS pathway, which involves transport through lysosomes or endosomes rather than the classical secretory pathway. Our findings of ECV release from hGFs is congruent with the notion of UPS as we found that Golgi inhibitors exerted minimal effects on ECV secretion, whereas inhibitors that target the UPS pathway markedly reduced ECV release. Accordingly, we searched for potential molecular partners that may contribute to UPS‐mediated ECV secretion. We identified AnxA2 as a key regulator of ECV secretion through mass spectrometry and co‐immunoprecipitation experiments. AnxA2, a Ca^2+^‐dependent phospholipid‐binding protein, facilitates the extracellular transport of specific proteins by inducing processes that include lipid segregation, membrane remodeling, and vesicle budding [32]. As AnxA2 lacks a signal peptide, it bypasses the conventional ER‐Golgi route and is thought to rely on direct translocation across the plasma membrane and vesicle‐related UPS pathway for secretion [54]. In response to stimuli such as IFN‐γ or temperature stress, AnxA2 translocates to the plasma membrane via an S100A10‐dependent process and subsequent release [55]. In this current study, we found an association between AnxA2 and vimentin by mass spectrometry and co‐immunoprecipitation and also found colocalization of these two proteins in the lamina propria of the periodontitis lesions. In cultured cells, this association was enhanced by inflammatory stimuli such as IL‐1β, which promotes the translocation of AnxA2 to the plasma membrane.

The association of AnxA2 with S100A10 stabilizes AnxA2 in a conformation that is conducive to phospholipid interactions, which is essential for these processes. Notably, our mass spectrometry data identified S100A10 as a vimentin‐binding protein, albeit at lower abundance compared with AnxA2. The AnxA2‐S100A10 hetero‐tetramer is essential for the translocation of AnxA2 from the intracellular to the extracellular environment and for its role in membrane remodeling and vesicle budding. We also found that AnxA2 knockdown reduces ECV secretion by > 70%. Collectively, these findings underscore the importance of AnxA2 in ECV secretion.

While previous data showed that serum levels of AnxA2 are higher in patients with periodontitis than in healthy controls [28], the role of AnxA2 in the pathogenesis of periodontitis is not defined. Our data indicate that the gingival inflammatory environment not only enhances AnxA2 abundance in the tissue but also promotes its association with vimentin, thereby facilitating ECV secretion. Elevated levels of ECV, in turn, may amplify the local inflammatory response, exacerbating periodontal tissue destruction. Further, AnxA2 can activate the NF‐κB signaling pathway [56] and promote plasminogen activation [57]. Collectively, these findings indicate a potential role for AnxA2 and vimentin in the amplification of the inflammatory response in periodontitis. Targeting the AnxA2‐dependent secretion pathway of ECV may provide a novel strategy to mitigate inflammation‐driven tissue damage in periodontitis. Further, AnxA2 and ECV expression levels could serve as biomarkers for disease progression and therapeutic efficacy.

In conclusion, our data on the secretion of ECV in the inflammatory microenvironment of periodontitis points to AnxA2 as a critical regulator of ECV transport. By integrating molecular mechanisms with a clinically relevant problem, such as control of progressive periodontitis, our data underpin the importance of elevated ECV levels in periodontal inflammation.

## Author Contributions

Z.Y. and Z.O.‐P. wrote the first draft of the manuscript. Z.Y. and C.A.M. wrote all subsequent versions of the manuscript with input from all other authors along the way. Z.O.‐P. and T.C. conducted cell culture, Western blotting, immunoprecipitation, mass spectrometry, and imaging experiments and analyzed the data statistically. M.N. analyzed the interaction of purified vimentin and AnxA2. A.A. performed the preparation, immunostaining, and imaging of human gingival specimens. Y.W. conducted the qRT–PCR assays. Molecular docking experiments were performed by M.A., I.J., and K.X. P.S. analyzed the abundance of vimentin in human GCF. All authors reviewed and approved the final manuscript version.

## Conflicts of Interest

The authors declare no conflicts of interest.

## Supporting information


Figure S1.

## Data Availability

Data sharing with interested parties will be conducted upon receipt of reasonable requests. All the mass spectrometry data (Table 1) are included in the text.
