# Supplementary material for: Annexin A2 Contributes to Release of Extracellular Vimentin in Response to Inflammation
Source: FASEB J. 2025 May 10;39(9):e70621. doi: 10.1096/fj.202500793R (PMC12065020; doi:10.1096/fj.202500793R)
Supplement: Supplementary file 1 — Figure S1. [file FSB2-39-e70621-s001.docx]

**Supplementary Figures**

**Supplementary Figure 1**


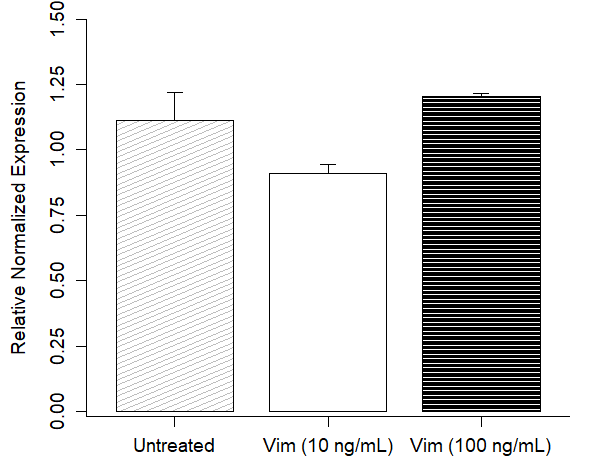


Human gingival fibroblasts (passage 22, hTert-immortalized, from Applied Biological Materials) were cultured on 60-mm² dishes at 5 × 10^5^ cells per dish overnight in complete DMEM. The next day, fetal bovine serum concentration was reduced from 10% to 1%. Cells were treated for 16 hours with either 10 or 100 ng/mL HA-tagged human Vimentin (expressed in E. coli using the pET-24 vector, with the 6× His tag replaced by an HA tag) in either 1 or 8 µL of PBS, or with 8 µL of PBS alone as a control. Total RNA was isolated from the cells; 1 µg of total RNA was reverse transcribed into cDNA. Amplification was performed using cDNA, sense and anti-sense primers. PCR cycling conditions were as follows: initial denaturation at 95°C for 2 min, followed by 40 cycles of denaturation at 95°C for 5 seconds and annealing/extension at 60°C for 25 seconds. Data (means±standard error of the mean; n=3 separate experiments) were normalized to β-actin and analyzed using the 2^-ΔΔCT^ method. Primer sequences for human TLR4 are forward 5’-ATATTGACAGGAAACCCCATCCA-3’ and reverse 5’-AGAGAGATTGAGTAGGGGCATTT-3’; for β-Actin are forward 5'-CATGTACGTTGCTATCCAGGC-3' and reverse 5'-CTCCTTAATGTCACGCACGAT-3'.

**Supplementary Figure 2**


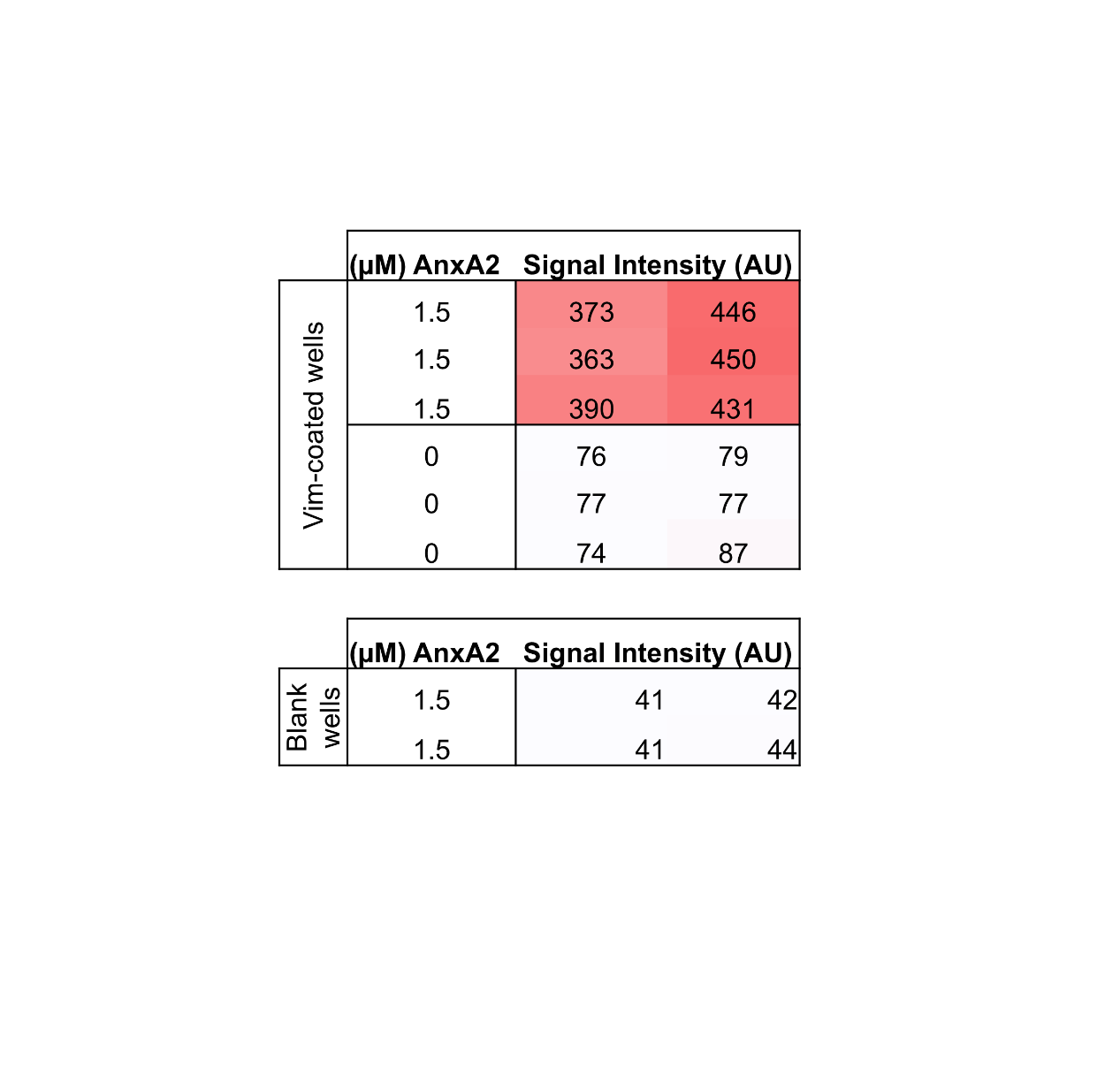


AnxA2-Vimentin protein interactions were quantified with an ELISA essay. Upper table: signal intensity of AnxA2 (1.5 µM) on a vimentin-coated substrate was 5-fold higher (p<0,001) than that of the 0 µM AnxA2 on vimentin-coated substrate. Lower table: signal intensity of AnxA2 (1.5 µM) binding to a blank substrate.

**Supplementary Figure 3**


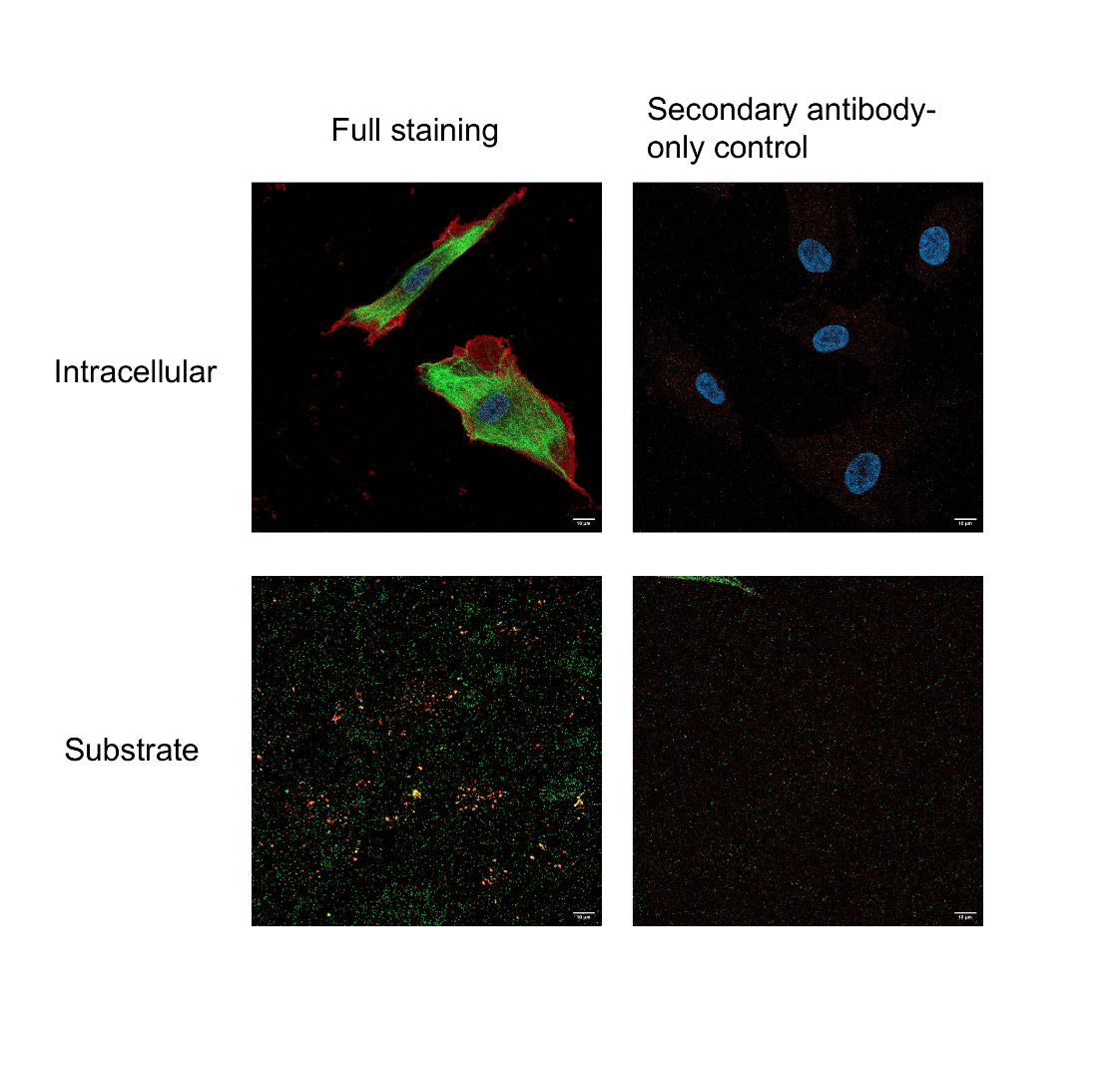


Immunostaining was performed for vimentin (green) and AnxA2 (red). Images were obtained of intracellular sites (top left panel; permeabilized cells) or of the cell-supporting substrate (lower left panel). These panels on the left show immunostaining with primary and secondary antibodies for vimentin and AnxA2 (designated as “Full Staining”). The panels on the right show permeabilized cells (upper) or the substrate (lower) stained with secondary antibody (control).
